# The HSP90 Inhibitor Ganetespib Alleviates Disease Progression and Augments Intermittent Cyclophosphamide Therapy in the MRL/lpr Mouse Model of Systemic Lupus Erythematosus

**DOI:** 10.1371/journal.pone.0127361

**Published:** 2015-05-14

**Authors:** Yuan Liu, Josephine Ye, Luisa Shin Ogawa, Takayo Inoue, Qin Huang, John Chu, Richard C. Bates, Weiwen Ying, Andrew J. Sonderfan, Patricia E. Rao, Dan Zhou

**Affiliations:** 1 Synta Pharmaceuticals Corp., Lexington, Massachusetts, United States of America; 2 Department of Pharmacology and Laboratory Medicine, VA Boston Healthcare System, West Roxbury, Massachusetts, United States of America; Instituto Nacional de Ciencias Medicas y Nutricion Salvador Zubiran, MEXICO

## Abstract

Systemic lupus erythematosus (SLE) is a complex, systemic autoimmune disease with a diverse range of immunological and clinical manifestations. The introduction of broad spectrum immunosuppressive therapies and better management of acute disease exacerbations have improved outcomes for lupus patients over recent years. However, these regimens are burdened by substantial toxicities and confer significantly higher risks of infection, thus there remains a significant and unmet medical need for alternative treatment options, particularly those with improved safety profiles. Heat shock protein 90 (HSP90) is a ubiquitously expressed molecular chaperone that acts as an important modulator of multiple innate and adaptive inflammatory processes. Of note, accumulating clinical and experimental evidence has implicated a role for HSP90 in the pathogenesis of SLE. Here we evaluated the potential of HSP90 as a therapeutic target for this disease using the selective small molecule inhibitor ganetespib in the well-characterized MRL/lpr autoimmune mouse model. In both the prophylactic and therapeutic dosing settings, ganetespib treatment promoted dramatic symptomatic improvements in multiple disease parameters, including suppression of autoantibody production and the preservation of renal tissue integrity and function. In addition, ganetespib exerted profound inhibitory effects on disease-related lymphadenopathy and splenomegaly, and reduced pathogenic T and B cell lineage populations in the spleen. Ganetespib monotherapy was found to be equally efficacious and tolerable when compared to an effective weekly dosing regimen of the standard-of-care immunosuppressive agent cyclophosphamide. Importantly, co-treatment of ganetespib with a sub-optimal, intermittent dosing schedule of cyclophosphamide resulted in superior therapeutic indices and maximal disease control. These findings highlight the potential of HSP90 inhibition as an alternative, and potentially complementary, strategy for therapeutic intervention in SLE. Such approaches may have important implications for disease management, particularly for limiting or preventing treatment-related toxicities, a major confounding factor in current SLE therapy.

## Introduction

Systemic lupus erythematosus (SLE) is a debilitating, systemic autoimmune disease characterized by loss of tolerance to nuclear self antigens, pathogenic autoantibody formation, immune complex deposition, and damage to multiple organ systems [1,2]. Clinically, SLE presents as a diverse and heterogeneous disease that follows an unpredictable yet unrelenting course involving flares and remissions. End-stage renal failure, cutaneous lesions, pulmonary fibrosis, neurological damage, and cardiovascular disease secondary to accelerated atherosclerosis represent primary complications for patients and contribute to the increased morbidity and mortality seen in this population [3]. It has been estimated that up to 1.5 million Americans currently suffer from SLE, the vast majority of whom tend to be young women during their reproductive years [4]. The chronic nature of SLE, its relapsing/remitting course, and cumulative organ damage over time presents a unique challenge to both patients and physicians alike.

Current treatment plans are typically dependent on the organs affected and disease severity—antimalarials and non-steroidal anti-inflammatory drugs are first-line treatments for mild-to-moderate disease; corticosteroids are commonly used to control flares; and immunosuppressants such as cyclophosphamide, methotrexate, azathioprine, and mycophenolate mofetil are prescribed to individuals with moderate-to-severe symptoms, often as steroid-sparing agents [5,6]. Unfortunately such broad-spectrum cytotoxic/immunosuppressive agents themselves exhibit substantial toxicities, may not adequately control disease symptoms, and inherently confer a greatly increased risk for infection [1]. Thus there exists a significant unmet medical need for alternate therapeutic options in SLE to improve patient outcomes and without the excessive toxicities of the current armamentarium of drugs.

Heat shock protein 90 (HSP90) is a ubiquitously expressed molecular chaperone that plays an essential role in normal cellular homeostasis by regulating the folding, stability and function of hundreds of cellular substrates, termed client proteins [7]. HSP90 is increasingly recognized as an important modulator of multiple innate and adaptive inflammatory processes [8] and, although a precise role in SLE is still undefined, a number of provocative findings have implicated HSP90 in the etiology of this disease. For example, increased levels of HSP90 have been observed in the peripheral blood mononuclear cell (PBMC) compartment of SLE patients, with elevated HSP90 expression correlating with enhanced levels of interleukin-6 (IL-6) and the presence of HSP90 autoantibodies [9]. In addition, the glomeruli of some SLE patients have been found to contain HSP90 deposits [10]. Of particular note, recent preclinical evidence suggests that selective HSP90 inhibitors may possess potential therapeutic utility for a number of inflammatory autoimmune diseases, including SLE [11–13].

Pharmacological blockade of HSP90 targets its clients for proteasomal degradation, in turn providing a means to coordinately impact multiple intracellular signaling cascades through one druggable target [7]. To date, targeted inhibition of HSP90 as a therapeutic strategy has predominantly been evaluated within the context of oncology [14,15] where it has emerged that small molecule HSP90 inhibitors may ultimately be best exploited in the clinical setting as combinatorial partners with standard-of-care agents [16]. In this manner they can act as chemotherapeutic sensitizers to provide improved efficacy while simultaneously reducing treatment-related toxicities. Here we provide a comprehensive evaluation of the efficacy of ganetespib, a potent and clinically advanced small molecule inhibitor of HSP90 [17], for improving disease outcomes in the well-characterized MRL/lpr mouse model of SLE [18]. Ganetespib is a fully synthetic, resorcinol-based compound that exhibits competitive binding with the N-terminal ATP-binding pocket of HSP90 in order to disrupt the chaperone cycle. The inhibitor is highly selective for HSP90, with favorable pharmacologic properties that distinguish the drug from other HSP90 inhibitors in terms of superior safety and tolerability [17]. Ganetespib was examined both as a single agent as well as in combination with cyclophosphamide in order to determine the comparative responses of lupus-forming animals to each of these treatment modalities. The findings of this study highlight the potential of HSP90 inhibition as an alternative, and potentially complementary, strategy for therapeutic intervention in SLE.

## Materials and Methods

### Ethics statement

All *in vivo* procedures were approved by the Synta Pharmaceuticals Corp. Institutional Animal Care and Use Committee and carried out in strict accordance with the Guide for Care and Use of Laboratory Animals of the National Institutes of Health. Animal euthanasia was performed using barbituate (sodium pentobarbital) overdose, and all efforts were made to minimize suffering. For the analysis of *ex vivo* cytokine production in human peripheral blood mononuclear cells, whole blood was collected from 5 healthy volunteers at Synta Pharmaceuticals who provided written informed consent. Protocol approval was provided by Aspire Institutional Review Board (Santee, CA) and the studies were conducted in compliance with the Declaration of Helsinki Protocols.

### Antibodies and reagents

Ganetespib was synthesized by Synta Pharmaceuticals Corporation. Cyclophosphamide monohydrate and lipopolysaccharide (LPS) were purchased from Sigma-Aldrich (St. Louis, MO), and CpG ODN 21798 from Miltenyi Biotec Inc. (San Diego, CA). Anti-human CD3 epsilon and CD28 antibodies were obtained from BD Biosciences (Franklin Lakes, NJ) and Ancell (Bayport, MN), respectively. CD3-APC, CD4-FITC, CD8-PE-Cy7, CD138-APC (BD Biosciences) and CD19-FITC, CD38-PE, B220-PE-Cy5.5 (eBiosciences, San Diego, CA) antibodies were used for flow cytometry. Antibodies for Western blotting, anti-AKT, ERK, BTK, IKKα and CAMKIV were from Cell Signaling Technology (Beverly, MA) and anti-LCK was from Santa Cruz Biotechnology (Dallas, TX).

### *In vitro* stimulation of PBMC and cytokine assay

Human PBMCs were isolated from heparinized peripheral blood on a Ficoll-Paque Plus gradient (GE Healthcare, Piscataway, NJ). Cells were suspended at 2 x 10^6^/ml in RPMI 1640 containing 10% FCS plus penicillin/streptomycin and seeded into 96-well plates. T cell stimulation was achieved using wells pre-coated with CD3 and CD28 antibodies (10 ug/ml in PBS) for 2 hours at 37°C. LPS (a TLR4 agonist) and CpG (a TLR9 agonist) were added to cell cultures immediately prior to drug addition at final concentrations of 0.5 EU/mL and 250 nM, respectively. Cells were dosed with dilutions of ganetespib (or dimethyl sulfoxide [DMSO] control) for 24 (LPS and CpG) or 72 hours (CD3+CD28). Supernatants were collected and cytokine levels measured using magnetic multiplex bead arrays (BioRad Laboratories, Hercules, CA, and Millipore, Bedford, MA). Cells stimulated with CD3+CD28 were tested for production of IL-2, IL-4, IL-5, IL-10, IL-12p70, IL-13, IL-17A, GM-CSF, IFNγ and TNFα. Cells stimulated through TLR4/9 were tested for production of IL-2, IFNγ, IL-10, IL-12p70, IL-1β, IL-6, IL-8, and TNFα. Each serial drug dilution was tested in duplicate using cells from 3–5 individual donors. Inhibition of cytokine production was calculated relative to DMSO controls and IC_50_ values determined by combining results from all individuals.

### Experimental animals and drug treatment

Female MRL/lpr mice, as well as non-lupus forming MRL/mp parental strain animals (Jackson Laboratories, Bar Harbor, ME), were maintained in a pathogen-free environment. For prophylactic dosing, beginning at 8 weeks of age, MRL/lpr mice were randomized into vehicle or ganetespib treatment groups (n = 2–3). Mice were i.v. dosed with vehicle or 50 mg/kg ganetespib formulated in DRD (10% DMSO, 14% Cremophor RH40, 3.8% dextrose in water) on a twice-weekly dosing schedule for 14 weeks. For the combination/therapeutic dosing studies, female MRL/lpr mice were allocated into treatment groups (n = 7–12) between 9 and 12 weeks of age following disease onset, as characterized by proteinuria scores ≥2. Animals were enrolled into groups with similar average dsDNA antibody titers (≥675). Antibody titers were established using the following methodology. OD_450_ values measured on a microplate reader (Dynex Technologies, Chantilly, VA) were plotted versus the reciprocal of the dilution factor and titers determined from linear regression analysis of that line using comparative OD_450_ values of serum samples from non-lupus MRL/mp mice run in the same assay. Five treatment regimens were examined: vehicle, 30 mg/kg cyclophosphamide i.p. 1x/week (CTX), 30 mg/kg cyclophosphamide 1x/2 weeks (CTX/2), 50 mg/kg ganetespib i.v. 2x/week, and the combination of ganetespib plus CTX/2. All animals (including untreated, age-matched non-lupus MRL/mp mice; n = 8) were sacrificed at 22 weeks of age.

### Pharmacokinetics

Plasma samples were collected from female MRL/lpr mice (n = 3) immediately prior to (time 0) and at 5 min, 1, 4, 6 and 24 h following the second intra-week dose of ganetespib while on study. Samples were extracted using a protein precipitation method by adding a 4X volume of methanol containing the internal standard. Samples were vortexed and centrifuged, and the resulting supernatants then analyzed by LC-MS/MS. A Phenomenex Kinetex 2.6μ C18 (30 x 2.1 mm) column was used with a run time of 3.5 min per sample. Pharmacokinetic parameters for ganetespib were calculated from plasma concentration data using the Noncompartmental Analysis module in Phoenix WinNonlin (version 6.3).

### Western blotting

Kidneys were harvested 6 and 24 hours following a second intra-weekly dose of vehicle (n = 3 animals/time point) or 50 mg/kg ganetespib (n = 4 animals/time point) to 8 week old MRL/lpr mice.

Renal tissues were pulverized using a CryoPrep Impactor (Covaris Inc., Woburn, MA) prior to extraction in lysis buffer (Cell Signaling Technology). Lysates were clarified by centrifugation and equal amounts of protein resolved by SDS-PAGE before transfer to nitrocellulose membranes (Invitrogen, Carlsbad CA). Membranes were blocked and then immunoblotted with antibodies directed against inducible Hsp70/72 (Enzo Life Sciences, Farmingdale, NY) or GAPDH (Santa Cruz Biotechnology, Inc., Santa Cruz, CA). The antibody-antigen complex was visualized and quantitated using the Odyssey system (LI-COR, Lincoln, NE).

Splenocytes prepared from spleens of MRL/mp mice were stimulated using plate-bound CD3 and CD28 antibodies as described above for 24 hours in the presence of vehicle (DMSO) or 100nM ganetespib. Cells were collected from culture, washed, and lysed in Extraction Buffer (Cell Signaling Technology) with addition of protease and phosphatase inhibitors. Lysates were clarified by centrifugation at 15000g for 20 minutes and protein content measured using a BCA assay (Pierce) and equal amounts of protein resolved by SDS-PAGE before transfer onto nitrocellulose membranes. Membranes were probed with antibodies detecting AKT, ERK, BTK, LCK, IKKα, and CAMKIV. Bands were visualized and quantified using the Odyssey system.

### Renal histology

For histological analyses, the kidneys from each animal were removed, fixed in 10% buffered formalin until processing. Multiple stains were used to determine histopathology changes, including hematoxylin and eosin (H&E), periodic acid—Schiff (PAS), and trichrome stains, using standard protocols. All stained sections were evaluated in a blinded fashion by an independent, board-certified medical pathologist for scoring purposes. The methodology used for renal histopathological assessment incorporated seven parameters as follows: glomerular deposits (defined as eosinophilic diffuse nodular thickening changes), hypercellularity, glomerular necrosis, tubular atrophy, intratubular casts, interstitial chronic inflammation, and fibrosis. Each was given a score of 0–3 for null/minimal (0), mild (1), moderate (2), or severe disease (3), respectively. In addition, IgG immunostaining was carried out in formalin-fixed paraffin-embedded renal tissues using an anti-mouse IgG antibody (Abcam) with HRP conjugate for detection, according to the suppliers’ protocol. The immunostained sections were evaluated as previously described [19]. For semi-quantitative scoring, immunoreactive deposits were assessed at low magnification in 4–5 randomly selected fields with a minimum of 125 glomeruli counted and scored (negative vs. positive) per animal, with the percentage of immunoreactive glomeruli per treatment reported.

### Measurement of proteinuria

Urine samples were collected weekly and tested for proteinuria by a standard semiquantitative test using Siemens Uristix dipsticks (Seimens Healthcare Diagnostics Inc., Tarrytown, NY). Results were quantitated per manufacturers’ instructions as follows: dipstick reading of 0 mg/dL = 0; Trace = 1; 30–100 mg/dL = 2; 100–300 mg/dL = 3; 300–2000 mg/dL = 4; and >2000 mg/dL = 5.

### Serological analysis

Serum was collected at start of treatment, at 15 weeks of age, and at the time of euthanasia (22 weeks) and samples stored at −80°C until analysis. Total serum IgG, IgG2b, and anti-chromatin antibodies (with respect to anti-nucleosome antibody expression) were analyzed using commercially available Mouse IgG (Innovative Research, Novi, MI), Mouse IgG2b (Abcam, Cambridge, MA) and QUANTA Lite Chromatin (Inova Diagnostics Inc., San Diego, CA) ELISA kits, respectively, according to manufacturers’ instructions. Serum levels of antibodies to double stranded DNA (dsDNA) were also determined by ELISA. Briefly, plates were coated overnight at 4°C with 50 μl of 2.5 μg/ml calf thymus DNA (Sigma-Aldrich). After washing and blocking, plates were incubated for 1 hour at 37°C with serial dilutions of serum samples obtained from each treatment. Plates were then sequentially incubated with goat anti-mouse Biotin-SP (Millipore) followed by HRP-conjugated streptavidin (R&D Systems, Minneapolis, MN), and finally peroxidase substrate was added for color development. OD_450_ values were measured on a microplate reader and plotted versus the reciprocal of the dilution factor. Antibody titers were established from linear regression analysis of that line using the OD_450_ of a serum sample from a non-lupus MRL/mp mouse used at a comparable dilution run in the same assay.

### Evaluation of lymphoid organs

At necropsy, the extent of lymphadenopathy and splenomegaly was assessed based on the weight of the enlarged lymph nodes (including submaxillary, thoracic, axillary, renal, and mesenteric) and the spleen, respectively. For flow cytometry, splenocytes were prepared as single cell suspensions. Following erythrocyte lysis in ACK lysing buffer (150 mM NH_4_HCl, 10 mM KHCO_3_, and 0.1 mM EDTA, pH 7.2; Gibco, Grand Island, NY), splenocytes were washed, counted, resuspended in PBS and immunostained with an antibody cocktail for identification of T cell subsets (CD3-APC, CD4-FITC and CD8-PE-Cy7) or B and plasma cells (CD19-FITC, CD38-PE, B220-PE-Cy5.5 and CD138-APC). Samples were analyzed using an LSR II flow cytometer with FACSDiva V6.1 software (BD Biosciences). Lymphocyte subset data were analyzed using one-way ANOVA with Sidak’s multiple comparison test.

## Results

### Ganetespib inhibits inflammatory cytokine production in *ex vivo* stimulated lymphocytes

To investigate the immunomodulatory potential of ganetespib, effects on cytokine production were evaluated using human peripheral blood mononuclear cells (PBMCs) following *in vitro* triggering via CD3/CD28 TCR (T-cell receptor), TLR9 (Toll-like receptor 9) or TLR4 (Toll-like receptor 4) stimulation. PBMCs were isolated from individual donors and the cultures stimulated by CD3 + CD28 activation, lipopolysacchiride (LPS) challenge, or cytidine—phosphate—guanosine (CpG) treatment. With low nanomolar potency, ganetespib strongly inhibited the secretion of a broad array of PBMC-derived cytokines, irrespective of the activating stimulus used (IC_50_ values, 2–7 nM; Table 1). Ganetespib displays minimal cytotoxic activity against PBMCs at concentrations up to 1 μM (S1 Fig) suggesting that the highly potent effects on cytokine production induced by these stimuli were a selective response to HSP90 inhibition and not due to loss of cellular viability.

**Table 1:** HSP90 inhibition by ganetespib blocks *in vitro* PBMC cytokine production induced by multiple stimuli.

Stimulus	Cytokine (production pg/ml)	IC_50_ (nM)*
*CD3/CD28*	IL-5 (109–1632)	2 ± 1.2
	IL-17A (177–1763)	2 ± 1.5
	IL-10 (165–973)	3 ± 1.2
	IL-13 (425–5861)	3 ± 1.2
	IL-4 (7–10)	4 ± 1.7
	IFNγ (16503–131924)	5 ± 1.2
	IL-2 (678–2503)	6 ± 1.3
	GMCSF (119–5779)	6 ± 3.0
	TNFα (7922–29951)	7 ± 1.2
*LPS*	IL-1β (3840–30472)	2 ± 1.1
	IFNγ (141–11000)	2 ± 1.2
	TNFα (2316–15590)	3 ± 1.1
	IL-6 (28968–31274)	5 ± 1.1
	IL-10 (638–7868)	5 ± 1.0
	IFNα2 (14–441)	5 ± 1.0
	IL-8 (167194–324730)	6 ± 1.1
	IL-12p70 (17–575)	6 ± 1.2
*CpG*	IFNγ (7–150)	2 ± 1.1
	IL-6 (50–339)	4 ± 1.2
	IL-8 (288–2610)	4 ± 1.2
	TNFα (27–266)	5 ± 1.1
	IL-10 (13–231)	6 ± 1.1

* Mean ± SE (CD3/CD28, n = 5; LPS/CpG, n = 3)

### Prophylactic treatment with ganetespib ameliorates renal damage in MRL/lpr mice

**Fig 1:**
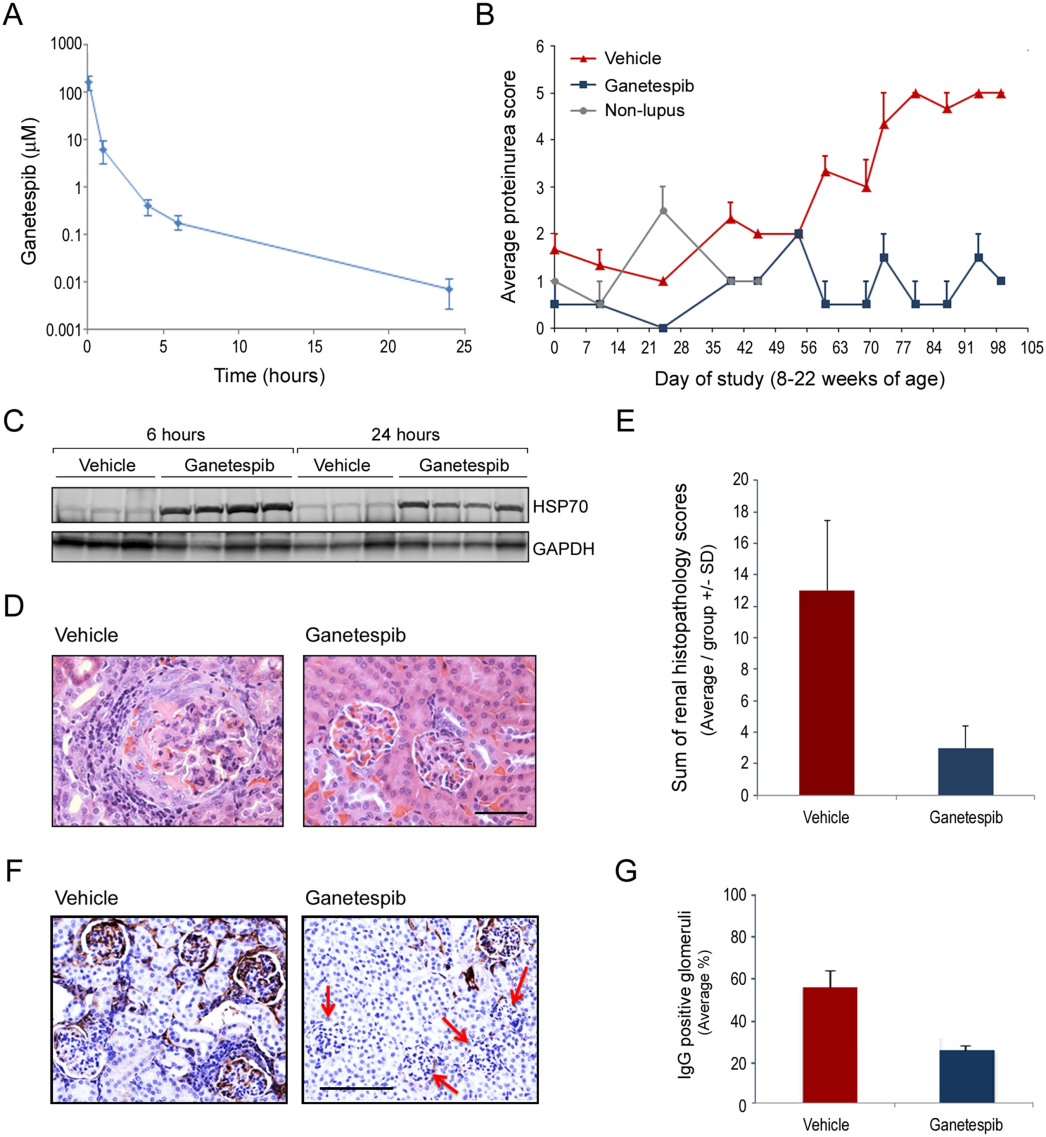
Ganetespib prevents the development of renal damage in a pilot prophylactic dosing study. (A) Mean pharmacokinetic plasma concentration-time profile after i.v. administration of ganetespib to MRL/lpr mice. Ganetespib was dosed at 50 mg/kg twice weekly and samples were taken after the second dose. Data represent mean ± SD (n = 3). (B) Average proteinuria scores (±SE) are plotted for vehicle and ganetespib-treated mice over the time course of the study. Average proteinuria scores for non-lupus parental strain (MRL/mp) mice from 8–15 weeks of age are included for comparison. (C) HSP70 induction as a pharmacodynamic marker of ganetespib activity in kidneys from drug-treated animals. Kidneys were harvested at 6 and 24 hours following the second intra-weekly dose of ganetespib (or vehicle) and lysates immunoblotted for HSP70 expression. GAPDH included as a loading control. Each lane represents renal tissue from an individual animal. (D) Representative H&E stained renal histopathology showing extensive chronic inflammation and thickening of the basement membrane with crescent formation in the glomerulus of a vehicle-treated mouse (*left panel*). In contrast, the kidneys of a ganetespib-treated animal showed no evident pathologic changes (*right panel*). Original magnification, 40X; scale bar represents 50 μm. (E) To evaluate total lupus-related renal damage 7 primary characteristics of GN (glomerular capillary deposits, hypercellularity, necrosis, tubular atrophy, intratubular casts, interstitial chronic inflammation, and fibrosis) were scored for each animal and summed for a composite disease score. The average summed pathology scores for each treatment group are plotted. (E) Representative IgG immunohistochemical staining of renal tissues harvested from vehicle- and ganetespib-treated animals at 22 weeks of age. Red arrows highlight the marked reduction in glomerular IgG deposition seen following HSP90 inhibitor treatment. Original magnification 20X: scale bar represents 100 μm. (F) Quantification of IgG glomerular immunoreactivity observed in vehicle and drug treated animals.

Next we performed a small-scale pilot study in order to determine whether ganetespib treatment would result in symptomatic improvement in the commonly used MRL/lpr mouse model of SLE. The MRL/lpr mouse exhibits a phenotype of systemic autoimmunity including immune complex-mediated glomerulonephritis (GN) that ultimately results in kidney failure, as well as increased IgG levels and autoantibody production [18]. Because disease onset in this model is relatively rapid, a prophylactic dosing regimen of ganetespib beginning at 8 weeks of age was initially investigated. Mice were dosed with ganetespib (50 mg/kg) twice weekly from 8 until 22 weeks of age. This dose level was selected based on favorable results in prior mouse tolerability studies, with the split-dosing regimen designed to provide optimal inhibition of the HSP90 chaperone protein. Pharmacokinetic analysis following the second consecutive ganetespib dose produced the plasma concentration-time profile shown in Fig 1A. The AUCt for ganetespib was 91.6 μM·h with a compound half-life of 3.6 hours.

A sensitive indicator of lupus-related GN is the presence of serum proteins in the urine (proteinuria) and this parameter is directly linked to both disease progression and degree of kidney damage. As shown in Fig 1B, proteinuria increased with age in vehicle-treated mice whereas ganetespib therapy effectively suppressed this symptom (to levels comparable to or below those observed in the non-lupus-forming, parental MRL/mp mouse strain). A conserved feature of pharmacological HSP90 blockade is triggering of the cellular heat shock response, characterized by upregulation of the stress-inducible chaperone HSP70 (heat shock protein 70) [20]. Accordingly, HSP70 induction is a commonly used pharmacodynamic biomarker of HSP90 inhibition and was therefore used as a biological readout for ganetespib activity *in situ*. HSP70 levels in the kidney were examined by immunoblotting following a second intra-weekly dose of either vehicle or ganetespib (Fig 1C). Robust induction of HSP70 was observed at both 6 and 24 hours post-dosing in ganetespib-treated animals, confirming that therapeutically relevant levels of the drug were present and active in this organ. Of note, upregulation of HSP70 in response to ganetespib exposure was also observed in spleen and PBMCs (data not shown), underscoring the systemic nature of the effects by this agent on additional SLE-related pathologies.

Consistent with the proteinuria findings, histological examination of renal tissues at week 22 revealed that ganetespib treatment significantly reduced the morphological appearance of GN in MRL/lpr mice, whereas vehicle-treated animals showed characteristic evidence of glomerular damage including hypercellularity, thickening of the basement membrane, and inflammation (Fig 1D). Using H&E, PAS, and trichrome staining, we evaluated 7 primary characteristics of GN: glomerular capillary deposits, hypercellularity, necrosis, tubular atrophy, intratubular casts, interstitial chronic inflammation, and fibrosis. Each disease feature was scored per animal and summed for a composite disease score. The average summed pathology scores for each group are presented in Fig 1E which showed that ganetespib exposure dramatically reduced the overall magnitude of renal damage. Extending these observations, the nature of the glomerular deposits was examined immunohistochemically using an antibody detecting mouse IgG (Fig 1F). Compared to vehicle-treated animals, ganetespib treatment markedly reduced the total number of IgG immunopositive glomeruli (quantitated in Fig 1G) but not the staining pattern. As shown in the S2 Fig, positive IgG staining featured dense dark brown, linear, nodular and diffuse changes in the glomeruli—consistent with the diffuse, nodular, eosinophilic patterns observed in the conventional H&E and PAS stained sections.

### Ganetespib treatment reduces serum anti-chromatin and anti-dsDNA antibody titers

**Fig 2:**
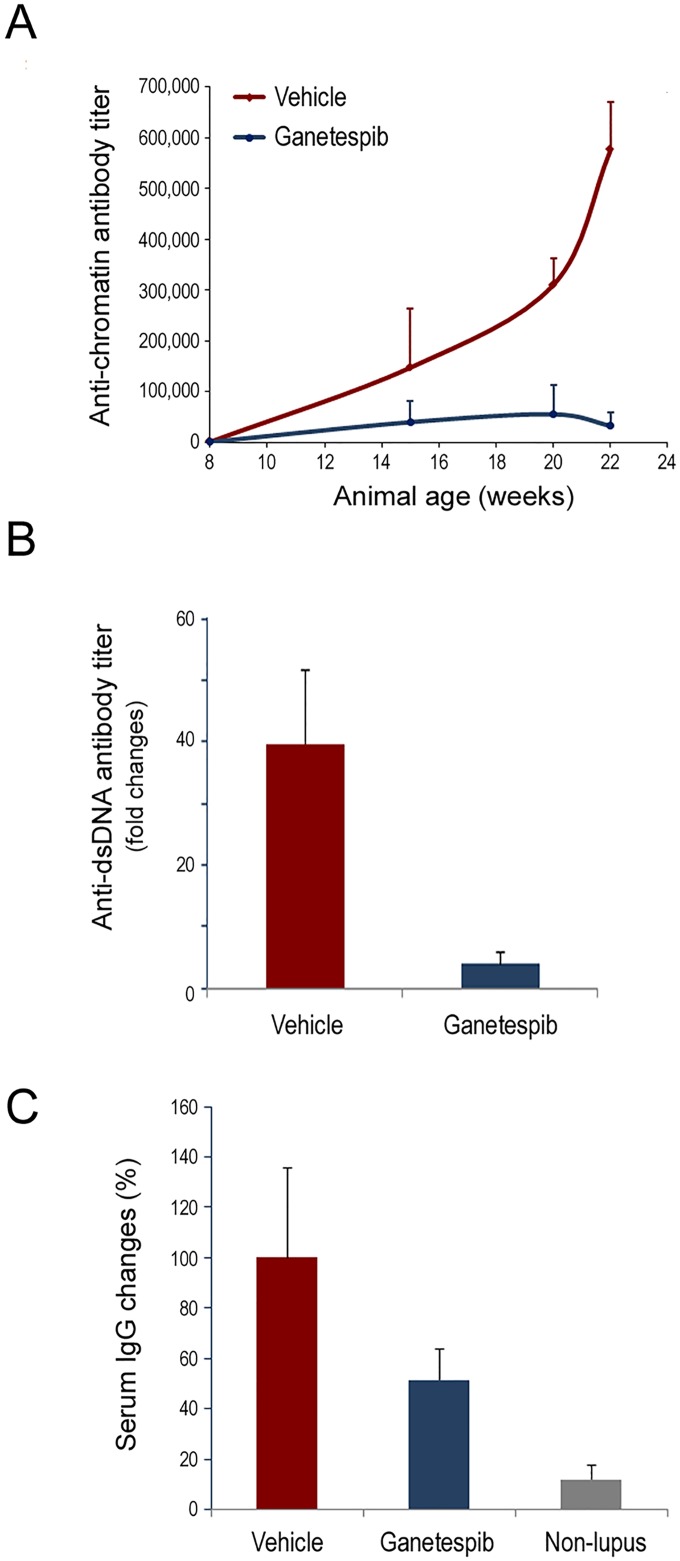
Ganetespib treatment suppresses autoantibody production. (A) Anti-chromatin autoantibody titers measured during twice—weekly dosing with vehicle or 50 mg/kg ganetespib for 14 weeks. Inverse titers are shown. (B) Fold-changes in circulating anti-dsDNA levels were determined following the 14 week treatment period. (C) Percentage changes in total serum IgG levels assessed after the 14 week treatment period. Serum changes in age-matched non-lupus MRL/Mp mice are included as a control.

The appearance and accumulation of anti-nuclear antibodies represent a hallmark of lupus and thus quantification of SLE-associated autoantibodies is a classical readout of disease progression. In vehicle-treated animals, serum anti-chromatin antibody levels increased significantly as a function of age, whereas ganetespib treatment normalized circulating levels over the same time course (Fig 2A). Similarly, anti-dsDNA autoantibodies were increased 40-fold in the vehicle group over the experimental period while ganetespib therapy suppressed this cumulative increase by greater than 90% (Fig 2B). In addition, these dramatic shifts in pathogenic autoantibody expression did not occur at the expense of overall serum IgG changes in ganetespib-treated animals, which were more modestly affected (approximately 50% reduction) following HSP90 inhibitor exposure (Fig 2C). Together with the renal damage findings above, these data show that ganetespib possesses robust single-agent activity and reduces multiple disease parameters in the MRL/lpr lupus model system, thus establishing the feasibility of using HSP90 inhibition as a treatment strategy for this disease.

### Ganetespib and cyclophosphamide exhibit similar therapeutic activity in attenuating glomerulonephritis progression in MRL/lpr mice

Having established this regimen of ganetespib as efficacious for disease control in the pilot study, a more robust assessment of this treatment modality compared to standard immunosuppressive therapy using cyclophosphamide was undertaken. Rather than prophylactic dosing, MRL/lpr mice were instead treated on a staggered therapeutic regimen following symptomatic onset of disease (between 9–12 weeks of age), as defined by a proteinuria score ≥2. Animals were allocated into treatment groups with similar average dsDNA antibody titers and treated with vehicle, twice weekly ganetespib (50 mg/kg), or weekly administration of cyclophosphamide at 30 mg/kg (CTX). The CTX dosing schedule was selected based on previously reported activity in this model [21]. While this experimental regimen is well tolerated in mice, cyclophosphamide therapy in human SLE patients is associated with a variety of adverse side effects. Therefore, we sought to determine whether ganetespib co-treatment could augment the activity of a less frequent cyclophosphamide dosing schedule in order to achieve similar or superior efficacies. To do this, additional groups of mice were treated with 30 mg/kg cyclophosphamide once every two weeks (CTX/2) either alone, or in combination with ganetespib therapy.

**Fig 3:**
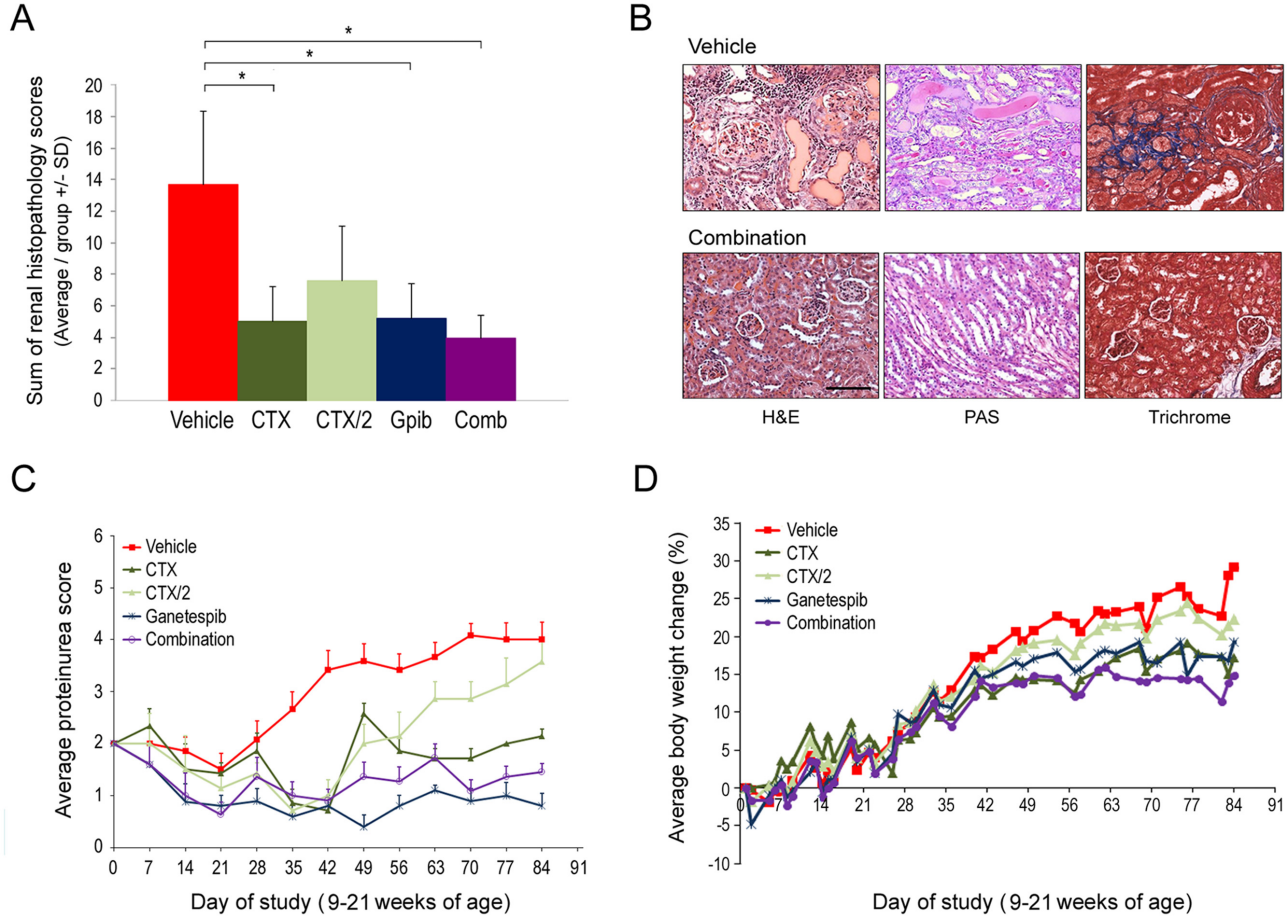
Comparative efficacy and tolerability of ganetespib and cyclophosphamide therapy in preventing glomerulonephritis progression using a therapeutic dosing regimen. (A) Composite renal disease scores. The average summed pathology scores for each treatment group are plotted (± SD). *p<0.05. *Gpib*, *ganetespib; Comb*, *combination*. (B) Representative renal histopathology from vehicle- and combination (ganetespib + CTX/2)-treated animals at the end of the study. In contrast to the kidneys from control animals which displayed marked glomerular crescent formation, chronic inflammation, tubular casts by PAS staining, and extensive fibrosis as evidenced by trichrome stain, combination treatment maintained near-normal glomerular and tubular architecture. Original magnification, 20X; scale bar represents 100 μm. (C) Serial measurements of urine protein were performed in individual mice and average proteinuria scores (±SE) are plotted for each treatment group. (D) Body weights were measured 3–4 times per week. Average percentage weight changes for each treatment group are shown.

Histological analysis revealed that CTX and ganetespib monotherapy each promoted similar and significant reductions in renal pathological scores compared to vehicle-treated animals (Fig 3A). As expected, less frequent cyclophosphamide dosing (CTX/2) mitigated the therapeutic response to this agent. However, when this sub-optimal regimen was combined with ganetespib treatment, the greatest overall reductions in renal damage were observed (Fig 3A), suggesting that targeted HSP90 inhibition adequately compensated for the diminished CTX immunosuppressive activity to ameliorate disease progression. Representative images of histostained kidney tissues harvested from vehicle- and combination-treated mice are shown in Fig 3B. The renal abnormalities observed in vehicle-treated animals with severe disease included focal glomerular hypercellularity, basement membrane thickening, chronic inflammation, glomerular atrophy with crescent formation, and prevalent intratubular casts. Tissue architectural integrity was preserved in mice treated with the ganetespib + CTX/2 regimen, with minimal evidence of any of these pathological changes detected by H&E or PAS staining. Similarly, when the extent of renal fibrosis was assessed by trichrome staining, the marked interstitial and periglomerular fibrosis observed in control animals was absent in combination-treated mice.

These findings were consistent with the proteinuria analysis (Fig 3C). The intermittent CTX/2 schedule delayed progression but was insufficient to provide durable disease control, with proteinuria scores increasing to levels comparable to control mice by the end of the study. In stark contrast, CTX dosing and both ganetespib-based treatments were efficacious in normalizing urinary protein excretion, thus providing clear evidence of disease stabilization. Importantly, all regimens (including combination drug therapy) were well tolerated, with no treatment-related losses of body weight seen over the course of treatment (Fig 3D), nor any significant clinical signs of toxicity observed. The higher body mass gains observed for vehicle- and CTX/2 treated animals were reflective of age-related disease progression in this model.

### Combination ganetespib plus intermittent cyclophosphamide treatment confers effective inhibition of autoantibody production

**Fig 4:**
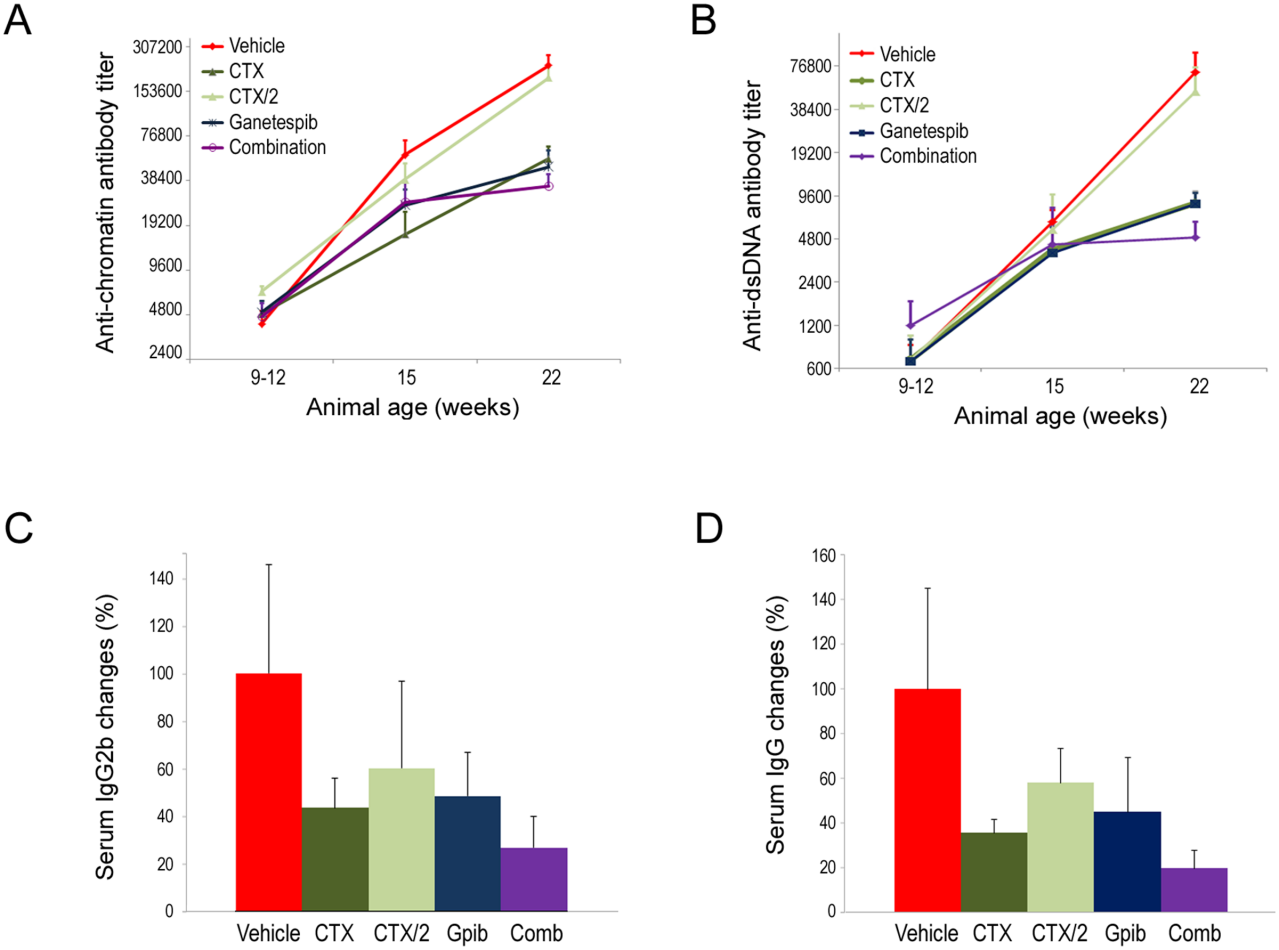
Combination ganetespib plus intermittent cyclophosphamide treatment effectively inhibits autoantibody production. Anti-chromatin (A) and anti-dsDNA (B) autoantibody titers were measured at three time points: upon initiation of therapeutic dosing (9–12 weeks of age); week 15; and at week 22. Average titers per treatment group (± SE) are presented. All treatment regimens except intermittent cyclophosphamide dosing alone (CTX/2) suppressed progressive age-related elevations in serum autoantibody levels. Inverse titers are shown. (C) Percentage changes in serum IgG2b levels (± SE) per treatment group. *Gpib*, *ganetespib; Comb*, *combination* (D) Percentage changes in total serum IgG levels (± SE) per treatment group. All reductions reached statistical significance compared to vehicle-treated control animals (p<0.05). *Gpib*, *ganetespib; Comb*, *combination*.

Next we performed serological analyses in mice undergoing each of the treatment regimens. In agreement with the findings above, ganetespib monotherapy and CTX treatment were similarly efficacious in limiting progressive age-related elevations in anti-chromatin and anti-dsDNA titers seen in vehicle-treated animals (Fig 4A and 4B). The lower cyclophosphamide dosing afforded by the CTX/2 schedule had no significant effect on circulating levels of either of these antibodies, yet the addition of ganetespib to this regimen resulted in maximal inhibitory effects on autoantibody production. This pattern was repeated when overall changes in serum IgG2b and overall IgG levels were quantitated (Fig 4C and 4D). For each case, combination therapy significantly reduced IgG2b and total IgG concentrations by 73% and 80%, respectively.

### Combination ganetespib plus cyclophosphamide treatment inhibits lymphoproliferation in MRL/lpr mice

**Fig 5:**
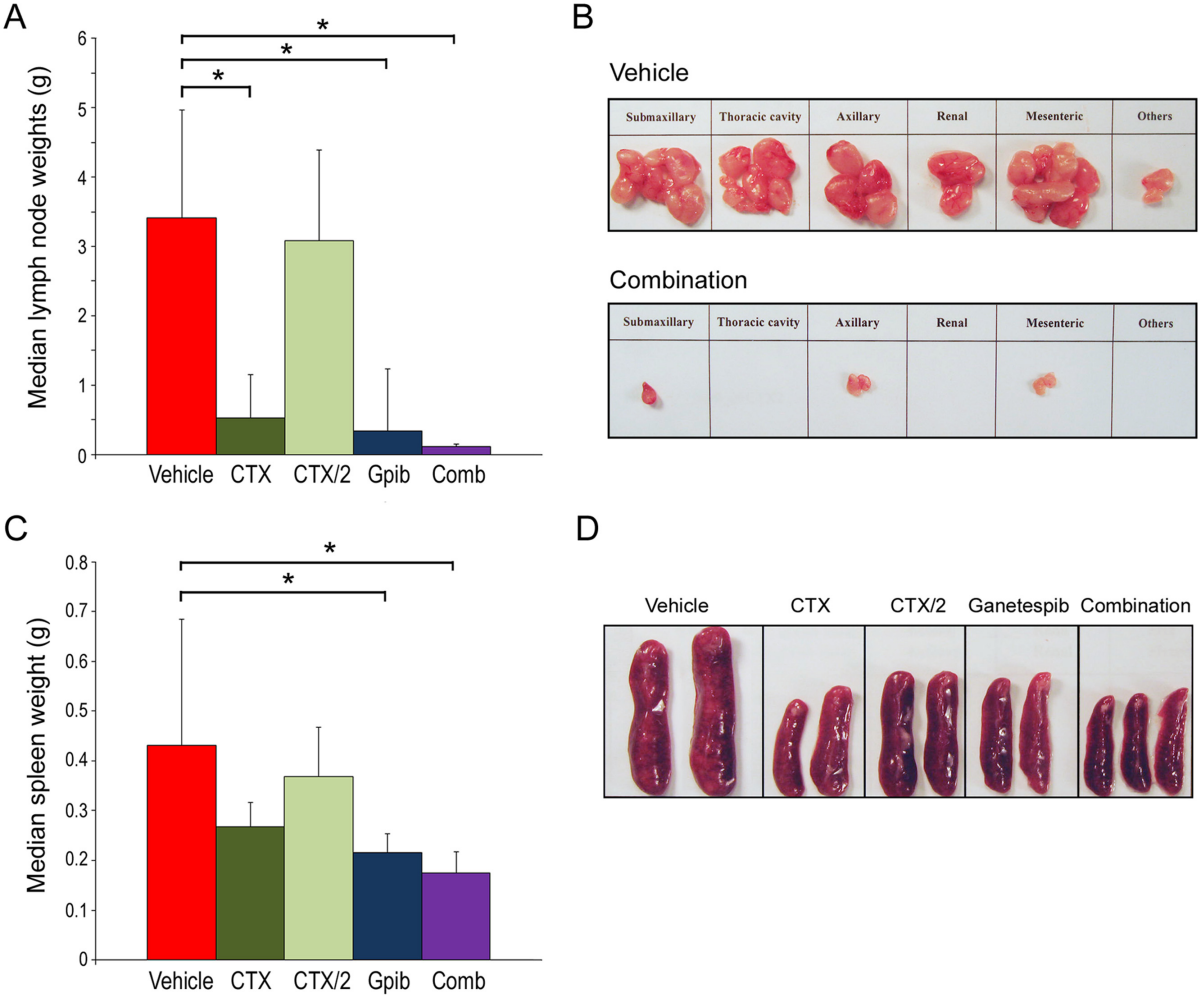
Absence of lymphoproliferation in combination-treated MRL/lpr mice. (A) Lymph nodes were harvested from mice upon completion of the individual therapeutic dosing regimens. Data are expressed as median pooled lymph node weights for each treatment (± SD). *p<0.05. *Gpib*, *ganetespib; Comb*, *combination*. (B) Representative images of regional lymph nodes harvested from vehicle- and combination (ganetespib + CTX/2)-treated animals at the end of the study. (C) Spleens were harvested at necropsy and weighed. Data are expressed as median spleen weights per treatment group (± SD).*p<0.05. (D) Representative images of spleens taken from mice from all treatment groups.

Lymphoproliferation is the hallmark of MRL/lpr mice and a characteristic disease feature in this model is massive lymphadenopathy associated with nodal accumulation of activated T cells [22]. Lymph nodes were harvested at necropsy and pooled weights were determined for all animals upon completion of treatment (Fig 5A). All regimens, with the exception of the sub-optimal CTX/2 dosing schedule, resulted in significant reductions in lymph node size when compared to those found in vehicle-treated animals that had undergone disease progression. Indeed, combination ganetespib + CTX/2 treatment completely abrogated lymphadenopathy *in situ*, resulting in an overall node weight burden indistinguishable from that observed for non-lupus animals (S3 Fig). Representative images of regional lymphatic tissues harvested from vehicle- and combination-treated mice are shown in Fig 5B.

Splenomegaly is another lymphoproliferative manifestation that arises in MRL/lpr mice. To determine the effects of CTX and ganetespib therapy on splenomegaly in these animals, spleens were collected and median organ weights compared for all treatment groups (Fig 5C). Cyclophosphamide treatment was moderately efficacious in reducing spleen weights, although neither dosing schedule reached statistical significance. However, and consistent with the lymph node findings above, spleen weights were more significantly affected in the single-agent ganetespib and combination-treated groups compared to vehicle controls. Interestingly, only modest, non-significant differences were noted between these two regimens, suggesting that maximal therapeutic response was being afforded by the HSP90 inhibitor itself. When spleen weights were expressed a percentage of body weight, a similar pattern and degree of symptomatic resolution was seen for each treatment (S3 Fig). Representative images of spleens harvested from animals undergoing all treatments are shown in Fig 5D.

### Therapeutic dosing regimens of ganetespib suppress the expansion of autoreactive T cells and abnormal plasma cells in the spleen

**Fig 6:**
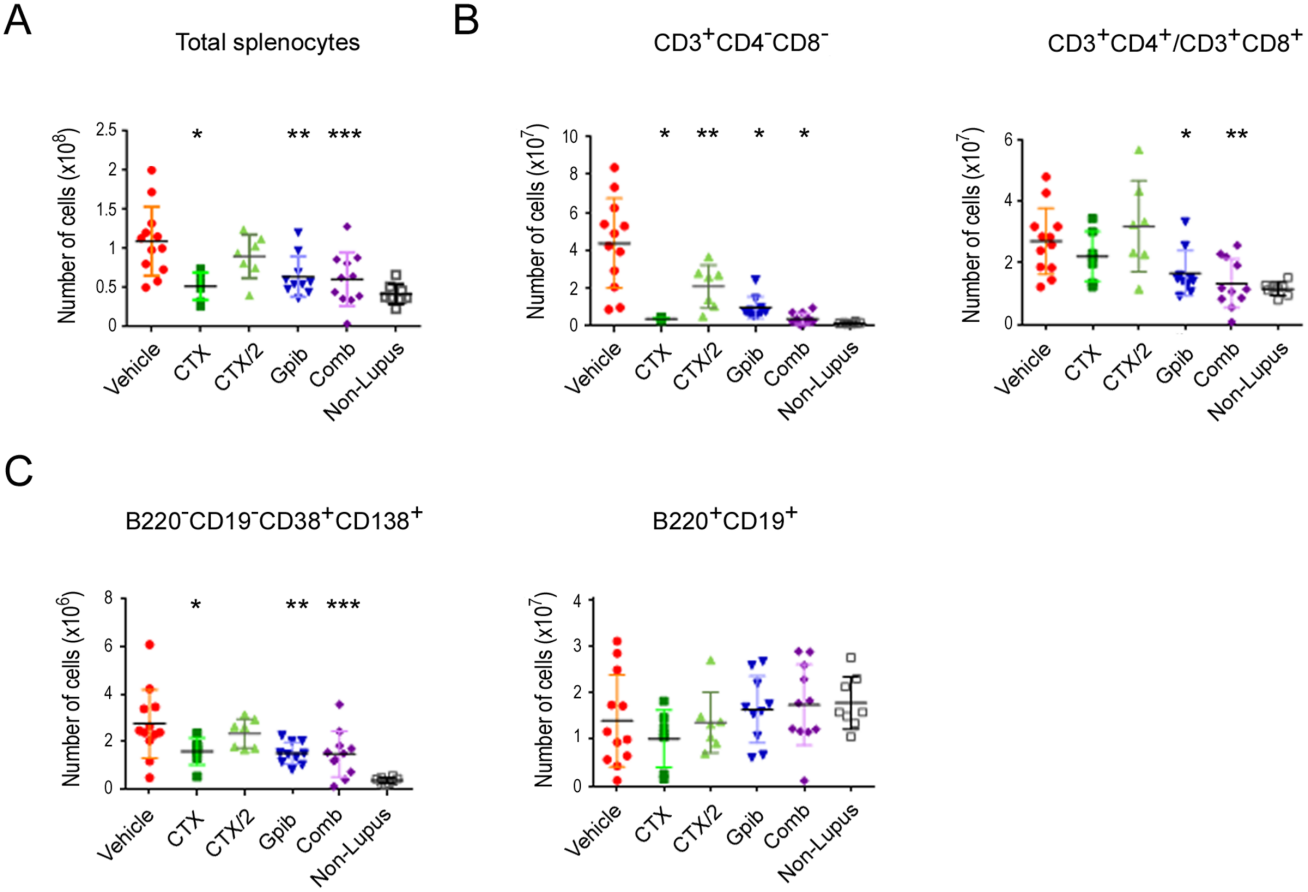
Ganetespib-based therapies suppress splenic expansion of activated T cells and abnormal plasma cells. (A) Plot shows the total number of splenocytes. Data are shown for 7–12 mice per treatment group. (*p<0.007; **p<0.0037; ***p<0.0012 versus vehicle-treated animals). (B) Splenocytes were isolated, counted and analyzed for the total number of CD3^+^CD4^-^CD8^-^ T cells (*left panel*; *p<0.0001, **p<0.0004) or the number of mature phenotype CD3^+^CD4^+^ and CD3^+^CD8^+^ T lymphocytes (*right panel*; *p = 0.04, **p = 0.002). (C) Numbers of B220^-^CD19^-^CD38^+^CD138^+^ plasma cells (*left panel*; *p = 0.03, **p = 0.007, ***p = 0.004) and B220^+^CD19^+^ B cells (*right panel*).

The lack of Fas receptor expression in the MRL/lpr mouse interferes with the deletion of activated mature T cells in the periphery, resulting in the expansion of large numbers of self-reactive T cells and the formation of CD3^+^CD4^-^CD8^-^ (double-negative) T cell populations [23,24]. Reactivity of these cells to self-antigens in lymphoid tissue further increases these populations to promote lymphadenopathy and splenomegaly, as well as supporting the differentiation and expansion of plasma cells responsible for autoantibody production. Therefore flow cytometric analysis was performed to quantify treatment-based effects on splenic lymphocyte subsets. Relative numbers of total splenocytes for each treatment group are presented in Fig 6A. Again, all regimens except CTX/2 dosing produced significant reductions in overall splenocyte numbers, indicating that effective cyclophosphamide and/or ganetespib therapy could modulate abnormal lymphocyte expansion in this model. As shown in Fig 6B, all treatments significantly suppressed the extensive accumulation of CD3^+^CD4^-^CD8^-^ T cells (left panel), with both CTX and combination therapy reducing this population to near normal levels. Interestingly, numbers of mature splenic T cells (CD3^+^CD4^+^/CD3^+^CD8^+^) were also elevated in response to disease progression relative to non-lupus animals (Fig 6B, right panel). Both single-agent ganetespib treatment and, in particular, combination therapy significantly reduced these populations to comparative levels seen in non-lupus mice.

With respect to B-cell lineages, we evaluated treatment effects on the abnormal plasma cell population characterized as B220^-^CD19^-^CD38^+^CD138^+^ (Fig 6C, left panel). This autoantibody-producing plasma cell subset was also sensitive to CTX, ganetespib, and combination ganetespib plus CTX/2 exposure, although reductions in absolute cell numbers were not as complete as those observed for autoreactive T cells. Finally, overall numbers of normal splenic B220^+^CD19^+^ B cells were not significantly different between mice of the MRL/lpr and non-autoimmune strains (Fig 6C, right panel). Importantly, this population was not significantly changed by any therapeutic regimen, underscoring the selective nature of the drug responses observed for the expanded cellular subsets.

### Ganetespib destabilizes kinases associated with T cell activation and disease development

**Fig 7:**
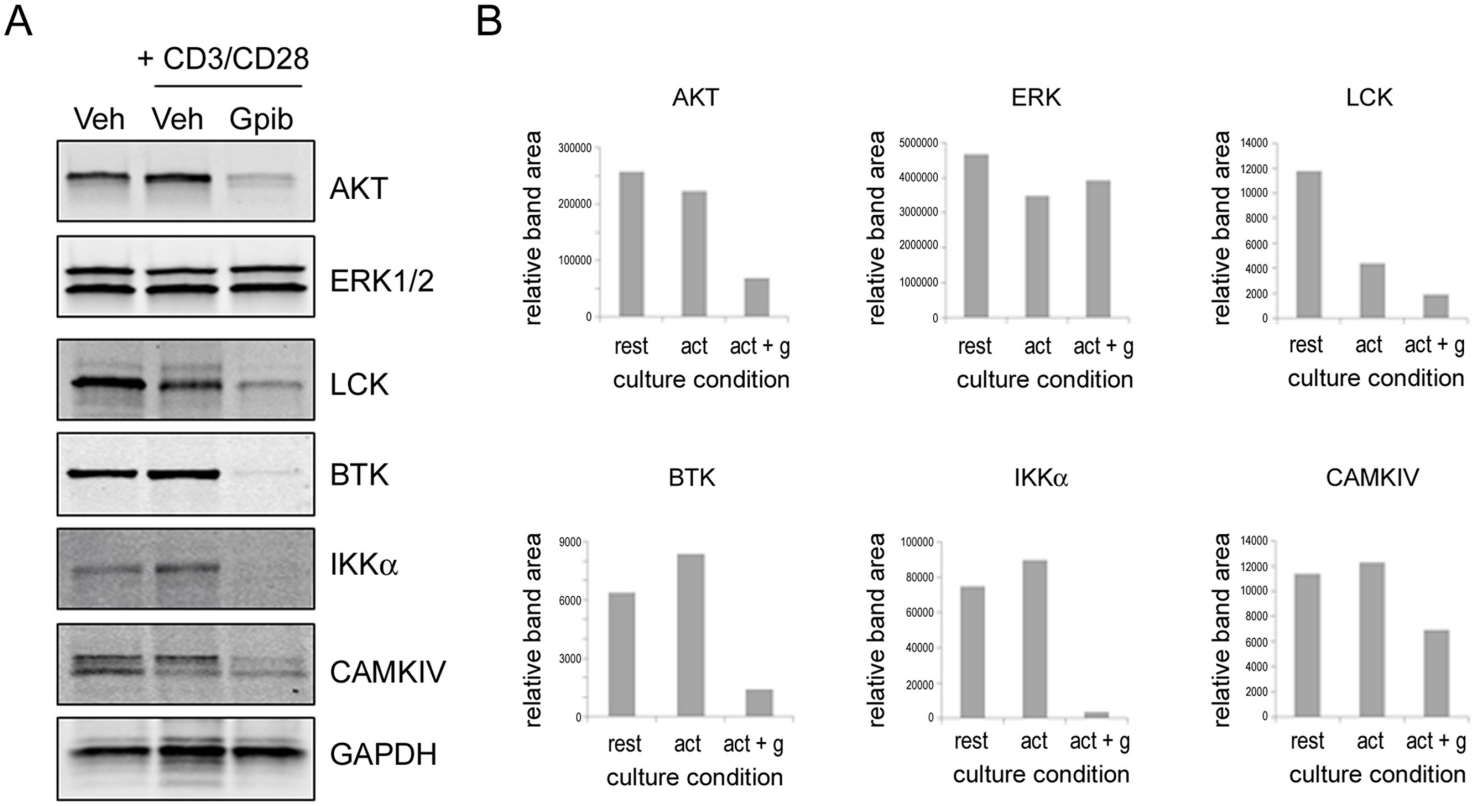
Ganetespib degrades kinases associated with T cell activation and disease pathology. (A) Splenocytes from MRL/mp mice were incubated for 24 hours with or without activation by plate-bound CD3 + CD28 in the presence or absence of 100nM ganetespib. Cells were lysed and levels of the indicated kinases were determined by Western blot. (B) Relative expression levels for each protein were normalized to GAPDH using densitomery. Culture conditions: *rest*, resting (unactivated); *act*, activated; *act + g*, activated plus ganetespib.

Towards a mechanistic understanding of ganetespibs’ activity, and because a number of kinases associated with lymphocyte activation are established HSP90 client proteins, finally we assessed the effect of ganetespib treatment on the stability of a number of kinases using splenocytes harvested from the parental strain. Splenocytes were prepared from MRL/mp mice and stimulated *in vitro* with CD3+CD28 in the presence or absence of 100nM ganetespib. After 24 hours, expression levels of kinases associated with T cell activation (AKT, ERK1/2, LCK) or reported to be associated with disease development in the MRL/lpr model (BTK, IKKα, and CAMKIV) [25–28] were examined by immunoblotting (Fig 7A). Both AKT and LCK were robustly destabilized following ganetespib exposure, while ERK1/2 expression levels remained unchanged. This lack of modulation was not unexpected, as ERK is not a direct HSP90 client and additionally serves as an important nodal effector of multiple upstream signaling cascades. Importantly, ganetespib treatment also resulted in degradation of the disease-related kinase set, most notably IKKα (Fig 7B), an effect which would be expected to effectively silence the NF-κB signaling pathway in these cells.

## Discussion

HSP90 exerts broad regulatory activity over many pro-inflammatory kinase cascades and plays important functional roles in processes that bridge innate and adaptive immune responses, including antigen presentation and lymphocyte maturation/activation [8,29]. Accordingly, pharmacological blockade of this chaperone using small molecule inhibitors has been shown to attenuate chronic inflammation and provide varying degrees of disease control in multiple inflammatory autoimmune disease models, including experimental autoimmune encephalomyelitis, rheumatoid arthritis, and uveitis [11,12,30–32]. For SLE, considerable experimental and clinical evidence has implicated a pathogenic role for HSP90, although a direct causative link remains to be established. Increased HSP90 expression and autoreactivity have been observed in SLE patients, and most frequently in individuals with active disease [33–35]. Elevations in other HSPs were not noted, suggesting that this phenotype was not merely due to non-specific HSP induction in response to disease-based stresses [36]. As a result, it has been postulated that SLE-related cytokine dysregulation can promote elevated HSP90 levels which, in turn, elicit the production of self-HSP90 autoantibodies. This model is supported by studies performed in transgenic mice as well as clinical observations [9,37], and the generation of anti-HSP90 antibodies observed in MRL/lpr mice matches disease onset and progression [38]. Further, high anti-HSP90 titers have been associated with renal disease [35] and, since the deposition of antigen-antibody complexes plays a significant role in immune-mediated damage to this organ, it is perhaps not surprising that glomerular deposits of HSP90 have been found in kidney biopsies of SLE patients [10]. Importantly, the presence of HSP90 in these renal deposits also appeared to be characteristic of SLE, since no HSP90 was observed in individuals with glomerulonephritis of non-SLE origin. In light of these considerations, here we explored the therapeutic potential of ganetespib, a selective HSP90 inhibitor currently undergoing clinical evaluation in multiple human oncology indications, for controlling SLE disease progression in MRL/lpr mice.

In addition to potent inhibition of cytokine production triggered via stimulation of the TCR and TLR receptors in human PBMCs, targeted HSP90 inhibition by ganetespib in isolated mouse splenocytes resulted in degradation of AKT and LCK, two kinases that play critical roles in T cell activation. Ganetespib treatment also resulted in effective destabilization of BTK and CAMKIV, small molecule inhibitors of which have previously been shown to reduce kidney damage and autoantibody production associated with MRL/lpr lupus-like disease [25–27]. Further, IKKα was strongly degraded following ganetespib exposure, thus providing a mechanism to abrogate NF-κB signal transduction. In this regard, inhibiting NF-κB activation using a novel, natural product inhibitor of NF-κB has been shown to reduce autoantibody production and skin lesions in MRL/lpr/lpr mice [28], although alone it was not sufficient to prevent kidney damage. Taken together, the multimodal and pleiotropic effects conferred by blockade of HSP90 suggest that ganetespib therapy offers a potential means to impact multiple organ systems damaged during the course of this disease.

In the prophylactic setting, twice-weekly administration of ganetespib (50 mg/kg) was highly efficacious in preventing the onset and development of the autoimmune lupus syndrome in these animals, based on a variety of pathological and serological readouts. It is interesting to note that this dosing level is considerably lower than that typically required for maximal antitumor activity in xenograft cancer models [39,40]. Specifically, ganetespib treatment preserved kidney tissue integrity, as evidenced by the marked reduction glomerular lesions and inflammation/immune-mediated damage that manifested in vehicle-treated animals. The concomitant prevention of proteinuria indicated that renal function was also protected in ganetespib-treated mice. In addition, ganetespib therapy significantly suppressed disease-dependent increases in circulating anti-chromatin and anti-dsDNA antibody titers. These findings are in agreement with those recently reported for MRL/lpr mice treated with the first-generation ansamycin HSP90 inhibitor, 17-DMAG [13]. In that study, however, disease control by 17-DMAG was comparatively less effective than the dramatic symptomatic improvements achieved with ganetespib. Ganetespib is structurally unrelated to the ansamycin class of HSP90 inhibitors and can be further distinguished from 17-DMAG in terms of superior potency and more favorable safety profile [41]. Overall, the high sensitivity of the MRL/lpr model to targeted HSP90 inhibition suggests that this therapeutic modality may represent a valid approach for the design of novel SLE treatments.

The beneficial effects of selective HSP90 blockade were also evident in the therapeutic dosing setting (i.e., initiated following disease onset) wherein a direct comparison of ganetespib treatment to standard-of-care immunosuppressive therapy was incorporated into the experimental design. Mice were administered cyclophosphamide (30 mg/kg) on a weekly schedule, a regimen previously reported as effective in improving the activity and chronicity index of nephritis in MRL/lpr animals [21]. Both treatment strategies were well tolerated and found to be equally efficacious in terms of disease control, exhibiting comparable degrees of renal protection and autoantibody suppression. In addition, ganetespib monotherapy exerted profound effects on the two major lymphoproliferative manifestations characteristic of this murine model (massive lymphadenopathy and splenomegaly) and more robustly than cyclophosphamide treatment alone. These data suggested that therapeutic HSP90 inhibition exerted durable antiproliferative effects within the lymphocyte compartment, sufficient to prevent the aberrant tissue accumulation of autoreactive T and B cells responsible for these pathologies. Of interest, this putative mechanism of action is distinct to that of cyclophosphamide, which acts through DNA damage and immunodepletion of these same target populations [42,43].

Both treatment modalities reduced total splenocyte numbers in MRL/lpr animals, including significant suppression of CD3^+^CD4^-^CD8^-^ T cell accumulation within this organ. Expansion of this ‘double-negative’ population plays a critical role in the pathophysiology of human SLE, in part via inappropriate tissue homing and organ infiltration, pro-inflammatory cytokine release, and triggering B cell antibody production [44,45]. Disease-related increases in the number of resident CD3^+^CD4^+^ and CD3^+^CD8^+^ mature T cells were also observed in the spleen. Notably, the aberrant accumulation of these lymphocyte populations was significantly suppressed by ganetespib, but not cyclophosphamide, treatment. Importantly, this therapeutic effect did not reduce overall numbers of these cells to below normal levels found in the spleens of non-diseased animals. Overall, it is now established that alterations in T cell differentiation and activity contribute to both the initiation and perpetuation of SLE [45]. Accordingly, targeting HSP90 function represents a logical point of intervention since HSP90 inhibition has been unequivocally shown to disrupt T cell activation, function, phenotype regulation, and stimulation of proinflammatory responses [46–48]. With respect to the dysregulated splenic population of abnormal plasma cells, significant reduction in numbers was achieved by either cyclophosphamide or ganetespib therapy. Interestingly, the number of B220^-^CD19^-^CD38^+^CD138^+^ plasma cells at the end of treatment remained substantially higher than those found in the spleens of normal, non-lupus mice. The presence of this elevated, residual population during the therapeutic time course suggested that the observed reductions in autoantibody titers primarily resulted from inhibition of antibody production at the cellular level and not through elimination of the long-lived plasma cell population. Similar effects have recently been described for targeted HSP90 blockade in mice with experimental epidermolysis bullosa acquisita (EBA) [49], another chronic autoimmune disorder characterized by pathogenic circulating and tissue-bound autoantibodies.

Immunosuppressant-based treatments used in the management of SLE, including pulse cyclophosphamide therapy, are burdened by significant short- and long-term side effects and life-threatening toxicities [50]. For example, in addition to a higher frequency of infections (often requiring hospitalization), cyclophosphamide use is also associated with bone marrow suppression, hemorrhagic cystitis, ovarian failure and infertility, and elevated risk of malignancy [6,50,51]. Thus there exists an inherent value in the design of new protocols that permit meaningful dose reductions of this powerfully immunosuppressive and cytotoxic agent that are sufficient to mitigate its toxicity profile yet maintain clinical effectiveness. Hence, a significant finding of the present study is the identification of a novel combinatorial strategy that produces therapeutic effects equivalent (or superior) to full cyclophosphamide dosing—achieved through combining ganetespib plus intermittent cyclophosphamide (CTX/2) treatment. While combination therapy consistently resulted in the greatest attenuation of glomerular damage as well as suppression of autoantibody production and lymphoproliferation, the observed differences between ganetespib monotherapy and combination treatment did not reach statistical significance in a study of this size. In addition to this limitation, two additional factors likely contributed to this result. First, the dosing level of ganetespib used was highly efficacious in its own right, and therefore likely masked the full extent of combinatorial benefit afforded by targeted HSP90 inhibition alongside immunosuppressive therapy. Second, the degree of symptomatic improvement achieved by ganetespib plus CTX/2 dosing in most cases resulted in post-treatment phenotypes that were indistinguishable from those of non-lupus animals, suggesting that no further biological improvement could be obtained. As part of its ongoing oncology clinical evaluation, over 800 patients have been treated with ganetespib to date. In these trials the compound has shown favorable long-term tolerability, with mild and manageable gastrointestinal toxicities representing the most common adverse events [52,53]. Unlike cyclophosphamide, ganetespib treatment for cancer has not resulted in bone marrow suppression. Therefore it is reasonable to suggest that the addition of a potentially potent and safe HSP90 inhibitor, such as ganetespib, to low-dose cyclophosphamide therapy would represent a rational approach to allow significant drug dose reductions of the more toxic cyclophosphamide with no loss of clinical effect. Based on the data presented here, we predict that this strategy could be optimized in order to simultaneously maintain therapeutic efficacy while minimizing unwanted side effects, and further exploration of this avenue of therapeutic intervention is warranted.

In summary, the dramatic responses to ganetespib-containing protocols observed in the MRL/lpr mouse suggest that pharmacological inhibition of the HSP90 chaperone pathway represents a potentially effective strategy for the treatment of SLE. An evolving understanding of the pathogenic mechanisms underlying SLE has resulted in the identification of new therapeutic targets for this disease, and a variety of biological agents targeting specific immune and inflammatory effector pathways are emerging as potential alternatives to the indiscriminate immunosuppressive and anti-inflammatory regimens currently in clinical use [6]. Due to the inherent heterogeneity of this disease, however, it is unlikely that any single, targeted approach would be effective for all patients and disease manifestations. In this regard, inhibiting HSP90 function represents a unique mechanism by which multifaceted impacts may be achieved across a spectra of disease symptoms, due to simultaneous and pleiotropic effects on multiple inflammatory and immunomodulatory processes using a drug with limited off-target toxicities. Overall, the findings presented here provide novel frameworks for the optimal design of HSP90 inhibitor-based therapies for the future management of SLE.

## Supporting Information

S1 Fig Ganetespib treatment is minimally cytotoxic to PBMCs. Human PBMCs were seeded into 96-well plates pre-coated with CD3 and CD28 antibodies and then incubated with either vehicle (DMSO) or graded dilutions of ganetespib (0.5–1000 nM) for 72 hours, Cells were harvested, stained with propidium iodide (PI) and viability assessed by flow cytometry. The percentage of PI-positive, non-viable cells are plotted as a function of drug concentration.
(TIF)

S2 Fig Glomerular IgG deposition. Representative glomerular IgG staining from a vehicle-treated MRL/lpr mouse at 22 weeks of age. The deposition pattern features prominent immunoreactivity consistent with eosinophilic diffuse nodular thickening changes in the glomerulus. Scale bar, 50 μm.
(TIF)

S3 Fig Resolution of lymphoadenopathy and splenomegaly with therapeutic dosing. (A) Lymph nodes were harvested from mice upon completion of the individual dosing regimens. Data are expressed as percentage of total body weight (± SD). *Gpib*, *ganetespib; Comb*, *combination; Non-L*, *non-lupus strain*. (B) Spleens were harvested at necropsy and weighed. Data are expressed as percentage of total body weight (± SD).
(TIF)
