# Supplementary figures and images for: The HSP90 Inhibitor Ganetespib Alleviates Disease Progression and Augments Intermittent Cyclophosphamide Therapy in the MRL/lpr Mouse Model of Systemic Lupus Erythematosus
Source: PLoS One. 2015 May 14;10(5):e0127361. doi: 10.1371/journal.pone.0127361 (PMC4431681; doi:10.1371/journal.pone.0127361)

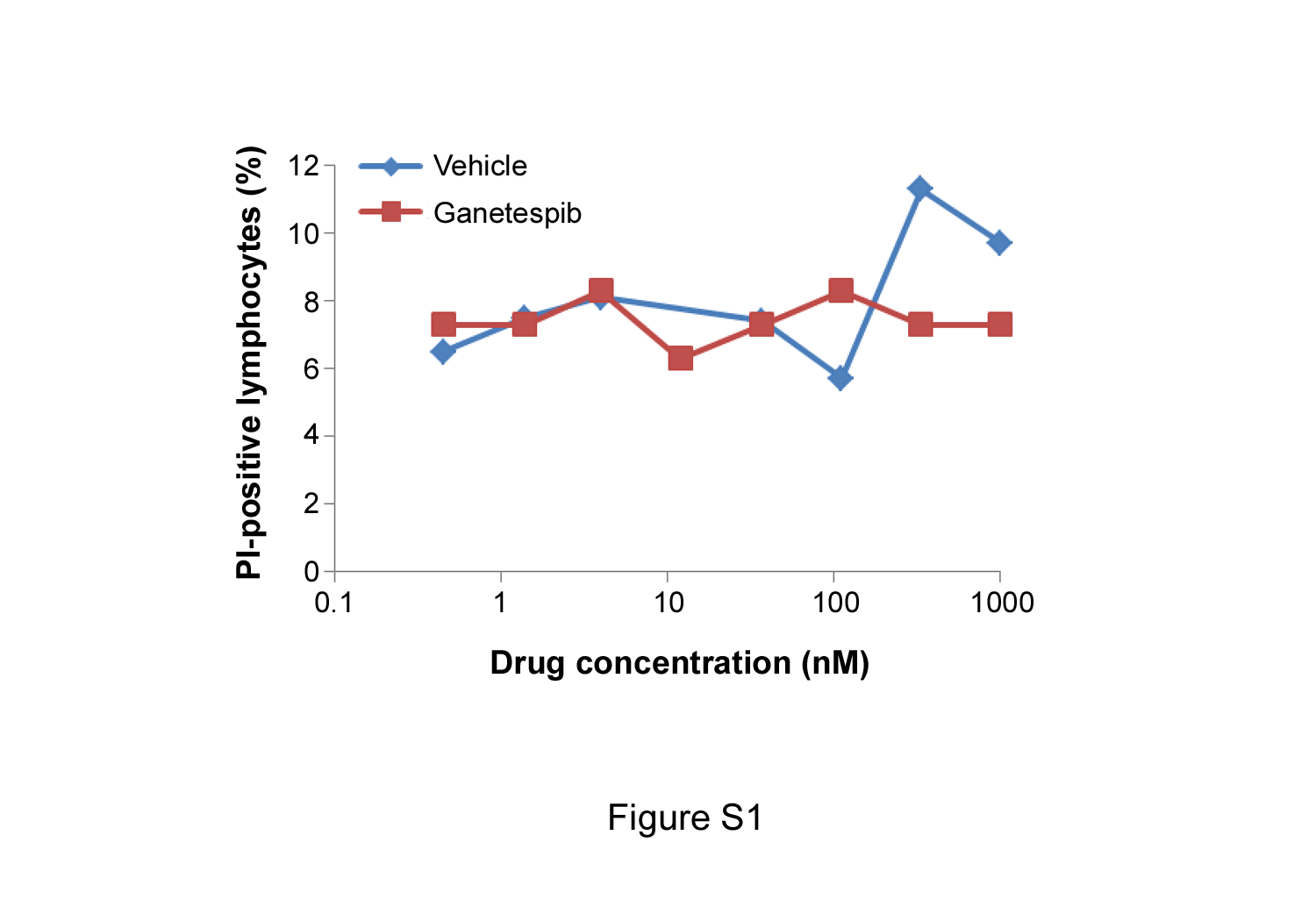

Supplement: S1 Fig — Human PBMCs were seeded into 96-well plates pre-coated with CD3 and CD28 antibodies and then incubated with either vehicle (DMSO) or graded dilutions of ganetespib (0.5–1000 nM) for 72 hours, Cells were harvested, stained with propidium iodide (PI) and viability assessed by flow cytometry. The percentage of PI-positive, non-viable cells are plotted as a function of drug concentration. (TIF) [file pone.0127361.s001.tif]

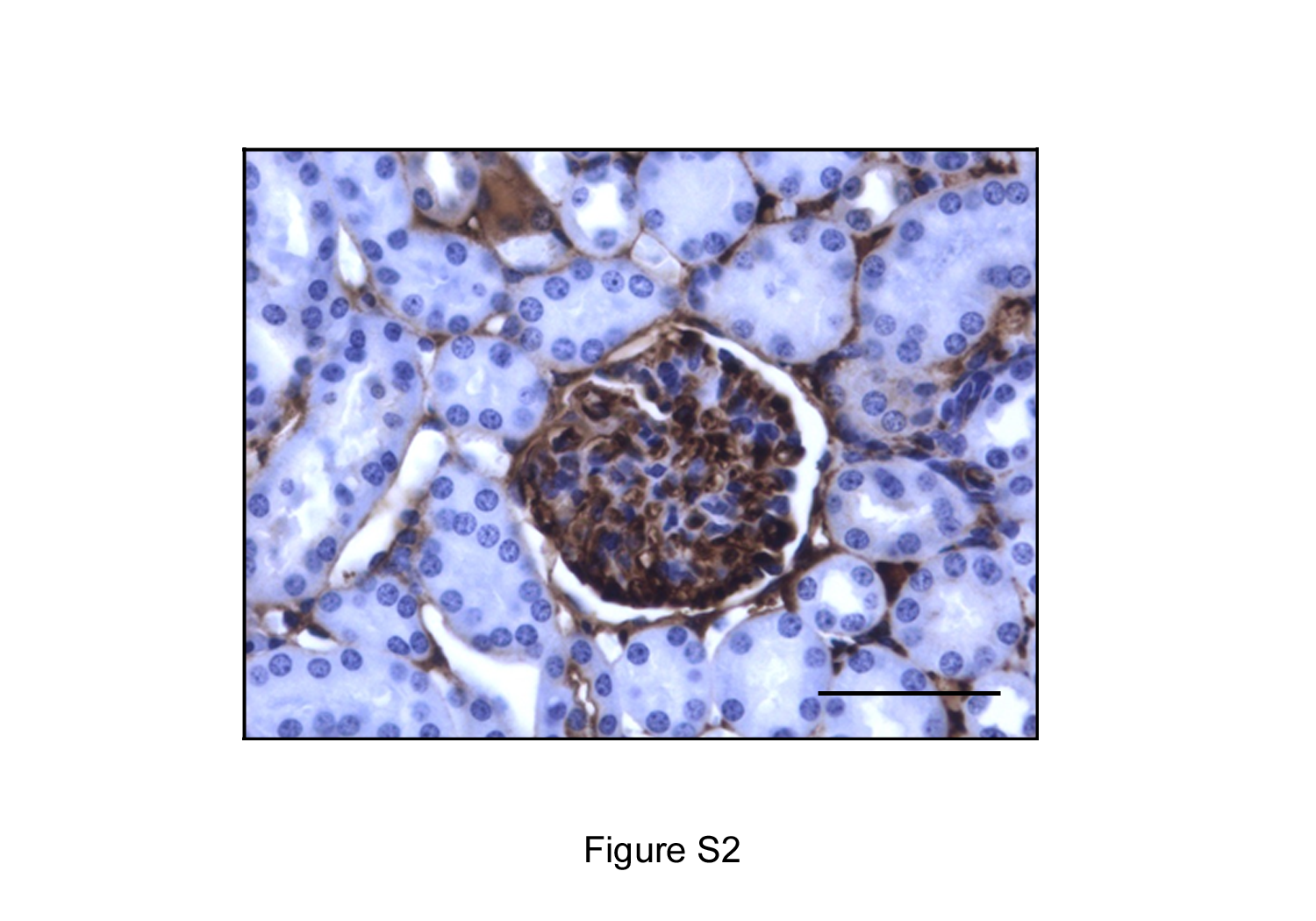

Supplement: S2 Fig — Representative glomerular IgG staining from a vehicle-treated MRL/lpr mouse at 22 weeks of age. The deposition pattern features prominent immunoreactivity consistent with eosinophilic diffuse nodular thickening changes in the glomerulus. Scale bar, 50 μm. (TIF) [file pone.0127361.s002.tif]

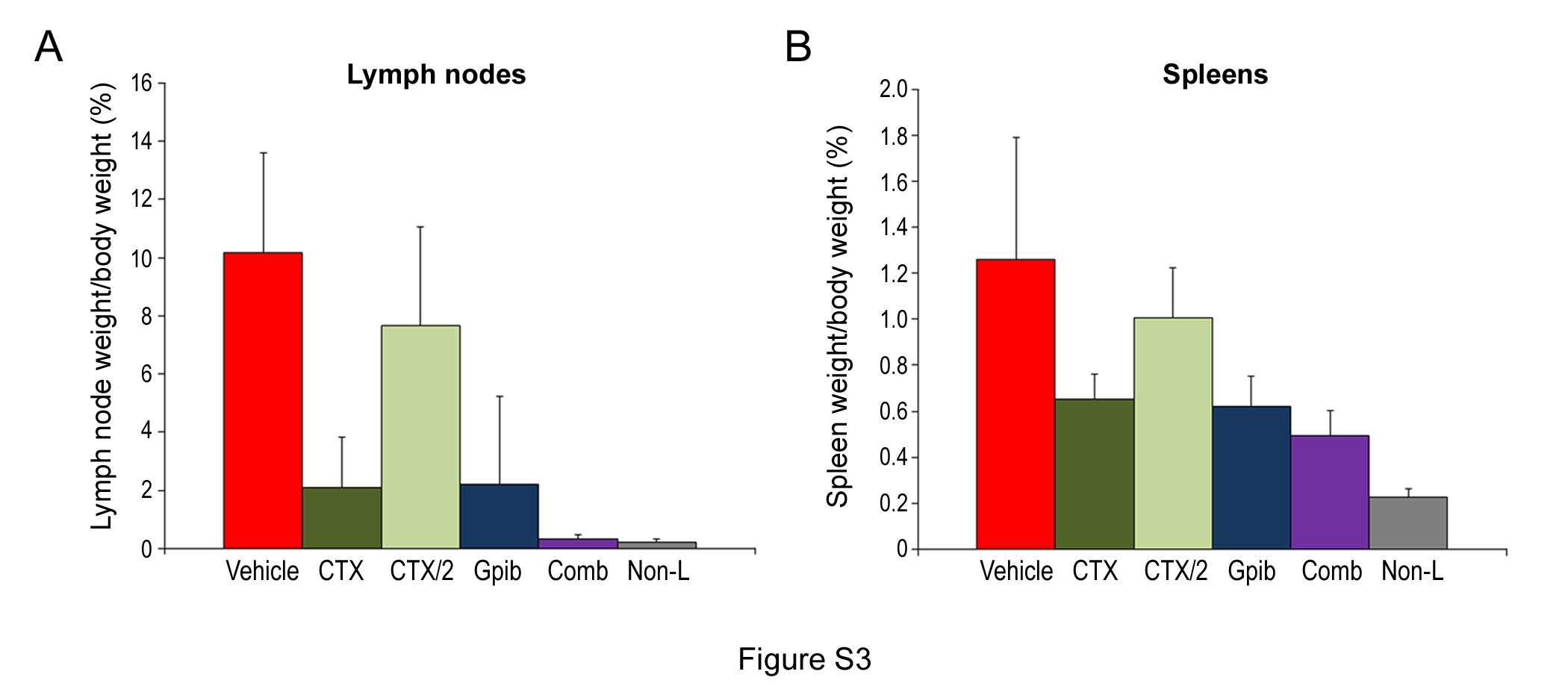

Supplement: S3 Fig — (A) Lymph nodes were harvested from mice upon completion of the individual dosing regimens. Data are expressed as percentage of total body weight (± SD). Gpib, ganetespib; Comb, combination; Non-L, non-lupus strain. (B) Spleens were harvested at necropsy and weighed. Data are expressed as percentage of total body weight (± SD). (TIF) [file pone.0127361.s003.tif]
